# Role of two sequence motifs of mesencephalic astrocyte-derived neurotrophic factor in its survival-promoting activity

**DOI:** 10.1038/cddis.2015.371

**Published:** 2015-12-31

**Authors:** K Mätlik, Li-ying Yu, A Eesmaa, M Hellman, P Lindholm, J Peränen, E Galli, J Anttila, M Saarma, P Permi, M Airavaara, U Arumäe

**Affiliations:** 1 Program in Developmental Biology, Institute of Biotechnology, University of Helsinki, Viikki Biocenter, PO Box 65, Helsinki 00014, Finland; 2 Program in Structural Biology and Biophysics, Institute of Biotechnology, University of Helsinki, PO Box 65, Helsinki 00014, Finland; 3 Department of Biological and Environmental Sciences, Nanoscience center, University of Jyvaskyla, PO Box 35, FI-40014, Jyvaskyla, Finland; 4 Department of Chemistry, Nanoscience center, University of Jyvaskyla, PO Box 35, FI-40014, Jyvaskyla, Finland; 5 Department of Gene Technology, Tallinn University of Technology, Akadeemia tee 15, Tallinn 12618, Estonia

## Abstract

Mesencephalic astrocyte-derived neurotrophic factor (MANF) is a prosurvival protein that protects the cells when applied intracellularly *in vitro* or extracellularly *in vivo*. Its protective mechanisms are poorly known. Here we studied the role of two short sequence motifs within the carboxy-(C) terminal domain of MANF in its neuroprotective activity: the CKGC sequence (a CXXC motif) that could be involved in redox reactions, and the C-terminal RTDL sequence, an endoplasmic reticulum (ER) retention signal. We mutated these motifs and analyzed the antiapoptotic effect and intracellular localization of these mutants of MANF when overexpressed in cultured sympathetic or sensory neurons. As an *in vivo* model for studying the effect of these mutants after their extracellular application, we used the rat model of cerebral ischemia. Even though we found no evidence for oxidoreductase activity of MANF, the mutation of CXXC motif completely abolished its protective effect, showing that this motif is crucial for both MANF's intracellular and extracellular activity. The RTDL motif was not needed for the neuroprotective activity of MANF after its extracellular application in the stroke model *in vivo*. However, *in vitro* the deletion of RTDL motif inactivated MANF in the sympathetic neurons where the mutant protein localized to Golgi, but not in the sensory neurons where the mutant localized to the ER, showing that intracellular MANF protects these peripheral neurons *in vitro* only when localized to the ER.

The prosurvival proteins that actively keep the cells alive function as a counterbalance to the prodeath programs of the cell and are thereby essential players in morphogenesis and adult tissue homeostasis. Such survival-promoting proteins are also potential candidates for the treatment of pathological conditions, especially in the nervous system where the lost neurons are only rarely replaced by new ones. Some prosurvival proteins act extracellularly. For example, neurotrophic factors (NTFs) are secreted proteins that bind the cognate receptors on the surface of the cells, thereby triggering prosurvival signaling cascades.^1, 2, 3^ Other prosurvival proteins, such as Akt kinase, antiapoptotic Bcl2 family members, inhibitor of apoptosis (IAP) proteins and so on are not secreted and protect the cells intracellularly. A new family of survival-promoting proteins has recently been described^4^ that can act in both ways. This family consists of two proteins, mesencephalic astrocyte-derived neurotrophic factor (MANF)^5^ and cerebral dopamine neurotrophic factor (CDNF).^6^ Both factors, when delivered into the extracellular space of the brain or applied via viral vectors potently antagonize neurological damage in the rodent models of Parkinson's disease. MANF is also protective against cerebral ischemia^6, 7, 8, 9, 10^ and prevents degeneration of Purkinje cells in spinocerebellar ataxia.^11^ In these experiments, MANF and CDNF halted the death of the neurons and also stimulated regrowth of the dopaminergic fibers, acting thus as typical NTFs. On the other hand in non-neuronal cells, MANF was also shown to be a resident protein of the endoplasmic reticulum (ER) protecting the cells intracellularly against ER stress.^12, 13, 14, 15, 16, 17^

In line with its role in counteracting cell death, MANF promotes pancreatic *β*-cell proliferation and survival *in vivo*, and lack of MANF leads to chronic unfolded protein response (UPR) activation in pancreatic islets.^18^ We have also shown that microinjected intracellular MANF protects cultured sympathetic neurons of the superior cervical ganglion (SCG) against apoptosis-inducing toxins, whereas it does not protect or bind these neurons when applied to the culture medium.^19^ Thus, MANF can protect the neurons when applied extracellularly (at least *in vivo*) and intracellularly. Of note, MANF also has both intra- and extracellular activities on the pancreatic *β* cells.^18^ However, the mechanisms of intra- and extracellular action of MANF are currently not well defined.

Structurally MANF consists of amino- (N) terminal saposin-like domain and carboxy-(C) terminal SAP domain-like domain that are connected by a flexible linker.^19, 20^ From the amino-acid sequence and structure, two potentially functional motifs can be distinguished in the carboxy-terminal domain of MANF (C-MANF).^19, 20^ First, a conserved^21, 22^ CXXC motif (CKGC) was found in the loop connecting the helices *α*7 and *α*8 where two cysteines were connected with a disulfide bond. The CXXC motif is found in the active center of the enzymes of thiol-disulfide oxidoreductase superfamily.^23^ Second, a conserved RTDL sequence belonging to the class of KDEL-like ER retention signal^24^ is found at the very end of C terminus of MANF^19, 20^ and is shown to be required for its retrieval from the Golgi to ER.^15, 16, 25^ The role of the RTDL motif in the survival-promoting activity of MANF has not been studied.

In this study, we set up to investigate the importance of these motifs of MANF for its survival-promoting activity both in the intracellular *in vitro* as well as extracellular *in vivo* paradigms. We mutated the CXXC and RTDL motifs and tested the activity of the mutants by microinjecting plasmid DNAs encoding the mutant proteins into apoptotic sympathetic SCG neurons, our validated model for testing the neuroprotective bioactivity of MANF,^19, 21^ and sensory dorsal root ganglion (DRG) neurons. Subcellular localization of the MANF mutants was also studied in the same neurons. We show that the CXXC motif is critically required for the survival-promoting activity of MANF, as mutation of this motif prevents MANF from being neuroprotective in both types of neurons *in vitro* and also in the *in vivo* rat model of cerebral ischemia. In addition, we show, using the same *in vivo* model, that the RTDL motif is not required for the neuroprotective activity of extracellularly applied MANF. However, intracellularly the mutant without RTDL motif was inactive only when it was not retrieved to the ER.

## Results

We mutated the identified motifs on human MANF and studied the survival-promoting properties of the mutants in comparison with wild-type (wt) MANF on three neuronal models that represent the two known modes of action of MANF. The intracellular prosurvival activity and subcellular localization of the mutants were studied in the models of apoptotic primary SCG and DRG neurons, the former being currently the best-characterized model to study neuronal survival.^26, 27, 28^ As MANF also protects the neurons when injected into the extracellular space of the brain tissue, for example, on the model of cerebral ischemia,^9, 10^ we studied the prosurvival activity of MANF mutants on that model as well.

### Mutation of CXXC motif inactivates intracellular MANF but does not change its localization

**Figure 1:**
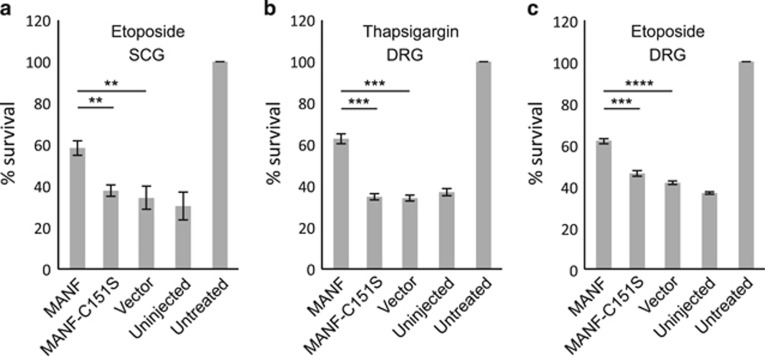
Mutation of cysteine 151 inactivates MANF. (**a**) NGF-maintained SCG neurons were microinjected with the indicated expression plasmids and treated with 20 *μ*M etoposide (*n*=3). (**b**–**c**) NGF-maintained DRG neurons were microinjected with the indicated expression plasmids and treated with 500 nM thapsigargin (**b**) or 20 *μ*M etoposide (**c**) (*n*=4 for both). The number of living injected neurons was counted 72 h later and is expressed as % of the number of original living injected neurons counted 3–4 h after injection. Fifty to eighty neurons were successfully injected per experimental group in each repeat. Vector denotes the empty plasmid pCR3.1. Shown are the means±S.E.M. Experimental groups were compared by one-way analysis of variance (ANOVA) and Tukey's multiple comparison *post hoc* test. **, *** and **** denote *P*<0.01, *P<*0.001 and *P<*0.0001, respectively. The null hypothesis was rejected at *P*<0.05

**Figure 2:**
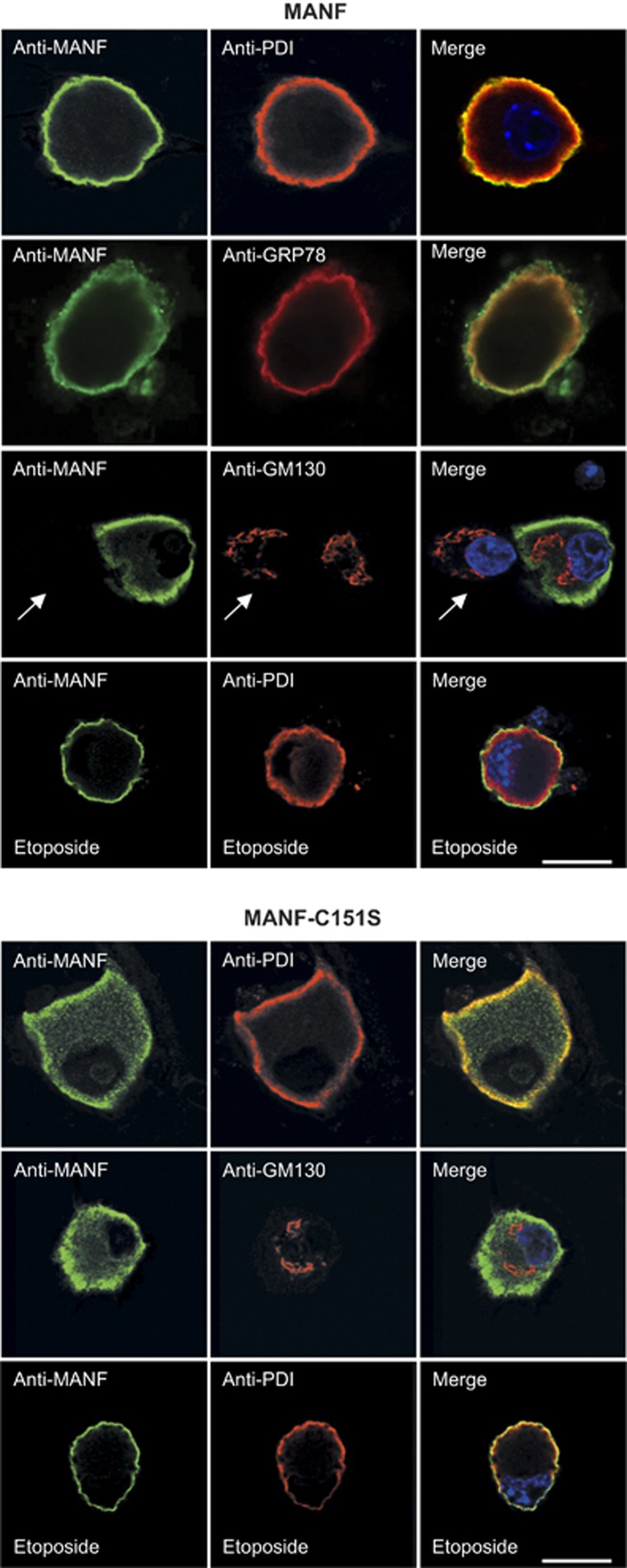
Immunolocalization of overexpressed MANF or MANF-C151S in healthy or etoposide-treated SCG neurons. NGF-maintained SCG neurons were microinjected with the expression plasmids for MANF or MANF-C151S and grown in the presence of NGF for 24 h or in the presence of NGF plus 20 *μ*M etoposide for 48 h. The cultures were costained with antibodies to MANF (green) and the ER markers PDI or GRP78 (red), or the Golgi marker GM130 (red). The nuclei were labeled with DAPI (blue). Shown are the confocal microscopic images of typical expression patterns. The MANF and MANF-C151S always colocalized with ER markers but not with GM130. Note the absence of MANF immunoreactivity in the uninjected neuron in the middle row of the MANF panel (arrow), showing the absence of nonspecific background and undetectable levels of endogenous MANF. Note the apoptotic fragmented nucleus in the etoposide-treated neuron that still has MANF in the ER. Scale bar, 12 *μ*m

In its C-terminal domain, MANF has a conserved sequence ^148^CKGC^151^ (amino-acid numbering of the mature protein).^19, 20^ To test whether this motif is essential for the survival-promoting function of MANF, we mutated cysteine 151 into serine (CKGC to CKGS) and overexpressed this construct (MANF-C151S) in the SCG neurons treated with etoposide, a cytotoxic drug that causes cell death by inhibition of topoisomerase II. The C151S mutation abrogated the survival-promoting activity of MANF almost completely (Figure 1a). C151S mutation had a similar inactivating effect on MANF's antiapoptotic activity in DRG neurons treated with etoposide (Figure 1c), and also with thapsigargin, an SERCA (sarco/endoplasmic reticulum Ca^2+^ ATPase) inhibitor that induces UPR and cell death (Figure 1b). It must be noted that the importance of CKGC motif for MANF's antiapoptotic effect could not be studied in SCG neurons treated with thapsigargin because thapsigargin did not kill the SCG neurons, even though it caused ER Ca^2+^ depletion and UPR (Supplementary Figure S1). To study the subcellular localization of MANF and its mutants, we coimmunostained the microinjected healthy NGF-maintained SCG neurons with antibodies to MANF and to either the ER markers protein disulfide isomerase (PDI) or 78 kDa glucose-regulated protein (GRP78), or to the Golgi marker GM130, and examined the neurons using confocal microscopy. In the cultured SCG neurons, the ER has an unusual localization at the peripheral submembrane area (Supplementary Figure S2). As shown in Figure 2, MANF colocalized with both ER markers and thus localizes mainly to the ER, as reported for other cells.^13, 14, 15, 16^ The level of endogenous MANF in the cultured sympathetic neurons was below the limit of detection, as revealed by immunostaining (arrow in Figure 2), although MANF mRNA was detected by RT-PCR in the SCG cultures (data not shown). MANF-C151S was also mainly located at the ER area, although in some neurons faint immunoreactivity was detectable in the areas away from ER (Figure 2). Both MANF and MANF-C151S colocalized with the ER marker PDI at the peripheral area also in cultured healthy DRG neurons (Supplementary Figure S3).

We also studied subcellular localization of overexpressed MANF and MANF-C151S in apoptotic neurons treated with etoposide for 48 h. The extent of death was estimated microscopically by morphological appearance of the neurons and the presence of condensed apoptotic chromatin in the nuclei, as revealed by 4',6-diamidino-2-phenylindole (DAPI) staining. In the healthy-looking neurons (either being protected by overexpressed wt MANF or resistant to etoposide at 48 h treatment), as well as in the neurons that were in the early stages of apoptosis, having condensed chromatin but not yet the other overt signs of apoptosis (shown in Figure 2), MANF was always located in the ER, mainly at its distal submembrane area (Figure 2). MANF-C151S was also located in the ER in the etoposide-treated neurons. We did not find any major relocalization of MANF and MANF-C151S from the ER to the cytoplasm or other organelles in the etoposide-treated neurons. All neurons in the advanced stages of apoptosis, injected or non-injected, showed strong general binding of anti-MANF antibodies that was most probably nonspecific (not shown).

**Figure 3:**
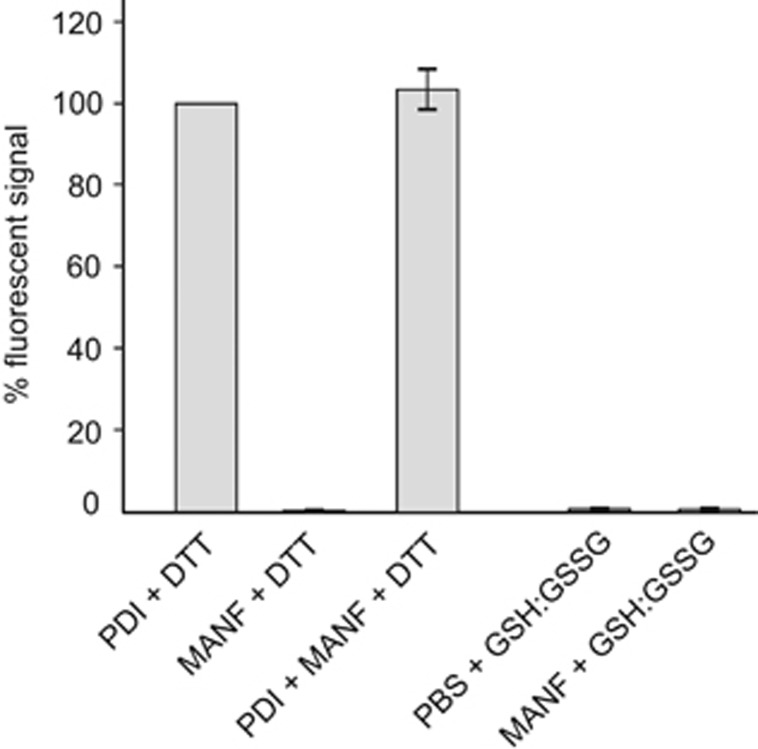
Inactivity of recombinant MANF in the insulin reduction assay. The ability of recombinant MANF protein (200 nM) to reduce insulin was tested using the Proteostat PDI Assay Kit. The activities of MANF and PDI, separately or together, were tested in the presence of reducing agents DTT or a mixture of reduced and oxidized glutathione (GSH/GSSG). The intensity of fluorescent signal from PDI-reduced insulin in the presence of DTT is taken as 100%. PBS was used as a negative control. Shown are the means of two independent experiments±S.E.M

The CXXC motif is found in the catalytic center of redox enzymes such as thiol-disulfide oxidoreductases, thioredoxins and glutaredoxins,^23^ suggesting that MANF could also function as a redox enzyme, and this catalytic activity could participate in its survival-promoting potency. To test whether purified recombinant MANF protein has any oxidoreductase activity on protein thiol groups, we used the widely used insulin reduction assay. As shown in Figure 3, 200 nM MANF did not catalyze insulin reduction in the presence of either dithiothreitol (DTT) or reduced glutathione, a more natural electron donor. Furthermore, MANF did not influence the insulin-reducing activity of PDI. Thus, no oxidoreductase-like activity of MANF was found in these assays.

In summary, our data suggest that the inactivity of C151S mutant MANF does not result from its improper localization or from the loss of its putative enzymatic activity (although enzymatic activity in other conditions cannot be excluded). This mutation may lead to subtle conformational changes or/and loss of interactions with other molecules.

### Deletion of C-terminal RTDL can reduce the intracellular survival-promoting activity of MANF by causing it to localize to the Golgi complex

**Figure 4:**
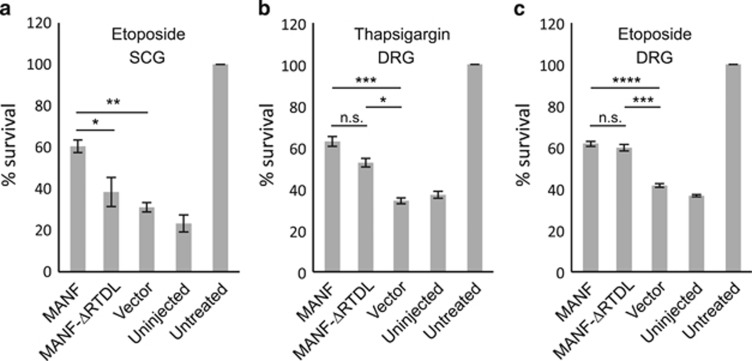
The effect of C-terminal RTDL deletion on the activity of MANF. (**a**) NGF-maintained SCG neurons were microinjected with the indicated expression plasmids and treated with 20 *μ*M etoposide (*n*=4). (**b**–**c**) NGF-maintained DRG neurons were microinjected with the indicated expression plasmids and treated with 500 nM thapsigargin (**b**) or 20 *μ*M etoposide (**c**) (*n*=4 for both). The number of living injected neurons was counted 72 h later and is expressed as % of the number of original living injected neurons counted 3–4 h after injection. Fifty to eighty neurons were successfully injected per experimental group in each repeat. Vector denotes the empty plasmid pCR3.1. Shown are the means±S.E.M. Experimental groups were compared by one-way analysis of variance (ANOVA) and Tukey's multiple comparison *post hoc* test. *, **, *** and **** denote *P*<0.05, *P*<0.01, *P<*0.001 and *P<*0.0001, respectively. NS, not significant. The null hypothesis was rejected at *P*<0.05

MANF has a conserved ^176^RTDL^179^ sequence in its C terminus, which belongs to the class of KDEL-like ER retention motifs.^16, 17, 19, 20, 24, 25^ To study the role of the RTDL motif in the survival-promoting activity of MANF, we deleted it and tested the mutant (MANF-ΔRTDL) in the etoposide-treated SCG neurons and DRG neurons treated with thapsigargin or etoposide. As shown in Figure 4a, in SCG neurons the survival-promoting activity of MANF-ΔRTDL was almost completely lost. However, deletion of the RTDL motif did not abrogate the protective effect of MANF in DRG neurons against either etoposide or thapsigargin (Figure 4b–c).

**Figure 5:**
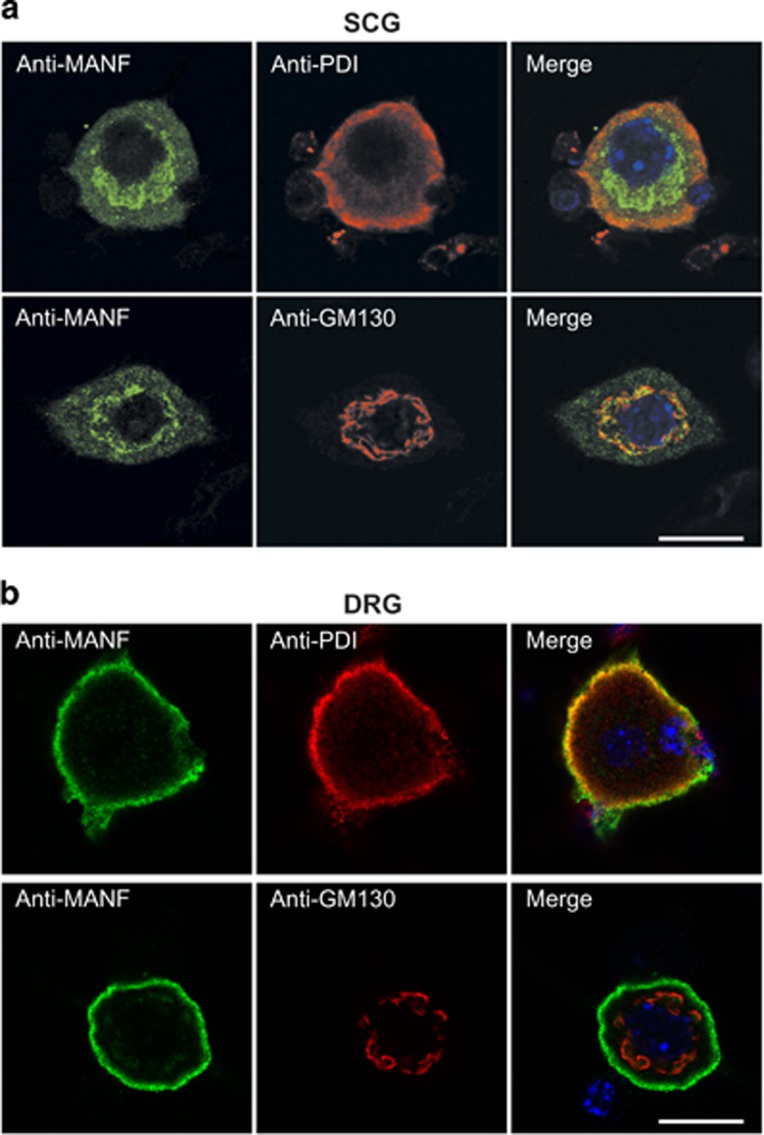
Immunolocalization of overexpressed MANF-ΔRTDL in SCG and DRG neurons. NGF-maintained SCG neurons (**a**) or DRG neurons (**b**) were microinjected with the expression plasmid for MANF-ΔRTDL, and 24 h later, the cultures were costained with antibodies to MANF (green) and PDI or GM130 (red), markers for ER and Golgi, respectively. The nuclei were labeled with DAPI (blue). Shown are the confocal microscopic images of the typical expression patterns. MANF-ΔRTDL localizes differently in these two types of neurons. Scale bar, 12 *μ*m

We studied the localization of RTDL-deleted proteins first in the microinjected SCG neurons. Notably, in contrast to MANF, in healthy SCG neurons the MANF-ΔRTDL mutant was prominently localized to the Golgi and only marginally to the ER (Figure 5a). Lack of RTDL sequence has been shown to increase the secretion of MANF.^15, 16, 17, 25^ We therefore used a sensitive enzyme-linked immunosorbent assay to test the levels of MANF and MANF-ΔRTDL in the culture media of SCG neurons 24 h after injection with the respective plasmids. We found that the secretion of MANF-ΔRTDL was slightly increased compared with wt MANF (Supplementary Figure S4).

We also studied the localization of MANF-ΔRTDL protein in healthy DRG neurons. Surprisingly, most of the MANF-ΔRTDL protein colocalized with the ER marker PDI, with only very weak immunoreactivity signal colocalizing with the Golgi marker GM130 (Figure 5b).

Thus, MANF without the RTDL motif was active when localized to the ER (in the DRG neurons), whereas in the SCG neurons most of the MANF-ΔRTDL cannot be retrieved from the Golgi to ER, which most probably results in the loss of its survival-promoting activity. Our results also show that in the DRG neurons, MANF can reside in the ER independently of the RTDL motif.

### The CXXC motif but not RTDL motif is required for neuroprotective activity of MANF in the rat model of stroke

**Figure 6:**
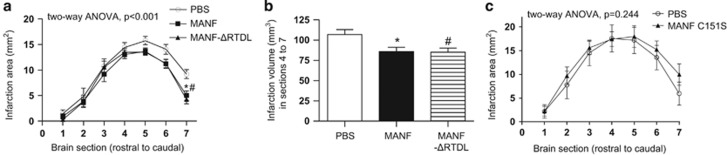
The CXXC motif, but not RTDL motif, is required for neuroprotective activity of MANF in the rat model of stroke. (**a**) Distribution of ischemic lesion along the rostrocaudal axis. Rats were injected with PBS (*n*=15), recombinant MANF (*n*=19) or MANF-ΔRTDL (*n*=18) and the MCA was occluded for 60 min. Two days later, the brains were cut into 2-mm-thick sections and stained with TTC. The average size of infarct is shown for each section, from the most rostral to the most caudal. *P<0.05 for MANF as compared with PBS, and ^#^*P*<0.05 for MANF-ΔRTDL as compared with PBS in Tukey's multiple comparison *post hoc* test following two-way analysis of variance (ANOVA). (**b**) The neuroprotective effect was confined to caudal portion of the brain. The infarction volume in brain sections 4 to 7 showed similar neuroprotective activity for both MANF and MANF-ΔRTDL. Differences between the treatments were determined by one-way ANOVA and Tukey's multiple comparison *post hoc* test. * and # denote *P*<0.05 of MANF and MANF-ΔRTDL, respectively, as compared with PBS using Tukey's multiple comparison *post hoc* test. The null hypothesis was rejected at *P*<0.05. (**c**) Rats injected with PBS (*n*=9) or recombinant MANF-C151S protein (*n*=11) underwent MCA occlusion for 60 min. Two days later, the brains were cut into 2-mm-thick sections and stained with TTC. The average size of infarct is shown for each section, from the most rostral to the most caudal. The distribution of ischemic lesion is not different between PBS- and MANF-C151S-injected groups (analysis by two-way ANOVA)

The experiments presented above revealed that both studied motifs are critical for the intracellular survival-promoting activity of MANF overexpressed in the SCG neurons, whereas in DRG neurons only the CXXC proved to be required for MANF's activity. MANF (and CDNF) can also protect the neurons extracellularly when injected into the brain.^6, 7, 8, 9, 10, 11, 29^ The mechanism of such extracellular survival-promoting activity *in vivo* is not yet clear. Recently, KDEL receptors that bind the RTDL motif were suggested to act as MANF receptors in the plasma membrane after thapsigargin treatment.^16^ We wished to use our MANF-ΔRTDL protein to test whether RTDL removal from MANF affects its neuroprotective effect in the rat model of focal cerebral ischemic injury. The rats were injected intracortically with purified recombinant MANF (*n*=19) or MANF-ΔRTDL (*n*=18) or phosphate-buffered saline solution (PBS) (*n*=15) and then underwent a 60-min middle cerebral artery (MCA) occlusion, followed by reperfusion, as described.^9^ Neuroprotective effects were assessed by determining the size of the ischemic lesion by triphenyltetrazolium chloride (TTC) staining two days after the surgery. The analysis of infarct area along the rostrocaudal axis revealed that MANF and MANF-ΔRTDL decreased the infarction area significantly as compared with the PBS-injected animals (Figure 6a, two-way ANOVA, treatment effect, F(2,363)=8.69, *P*<0.001). Bonferroni *post hoc* test revealed a significant treatment effect between PBS and MANF, and PBS and MANF-ΔRTDL (*P*=0.001 for both). The results of Tukey's multiple comparison test at each brain section are shown as asterisk and hashtag in Figure 6a. The neuroprotective effect was confined to caudal portion of the brain and analysis of infarction volume in the caudal sections 4 to 7 revealed a statistically significant difference between the treatments (Figure 6b, one-way ANOVA F(2,51)=4.77, *P*=0.013). The Tukey's multiple comparison *post hoc* test revealed a significant difference between PBS and MANF (*P*<0.05), and between PBS and MANF-ΔRTDL (*P*<0.05). Thus, MANF and MANF-ΔRTDL showed equal neuroprotection against ischemic brain injury. It should, however, be noted that the neuroprotective effect was smaller than reported previously with MANF,^9^ as the effect was confined to the caudal portion of the brain and there was no statistically significant difference in the total infarction volumes. However, protein-injected rats showed a clear tendency towards smaller lesion compared with the PBS-injected group (*P*=0.10, one-way ANOVA, the average±s.e.m. for PBS: 140.97±12.02 mm^3^; for MANF: 113.80±9.00 mm^3^; for MANF-ΔRTDL: 114.34±8.44 mm^3^). The smaller effect can be due to batch differences of recombinant protein production in *Escherichia coli*. We also tested the role of CXXC motif of MANF on the same model. Analysis of the infarct area along the rostrocaudal axis showed no difference between rats treated with MANF-C151S (*n*=11) and PBS-treated animals (*n*=9, Figure 6c, two-way ANOVA, *P*=0.244). We conclude that the C-terminal KDEL-like motif, which is critical for the intracellular neuroprotection by MANF in SCG neurons, is not required for its neuroprotective activity when applied extracellularly *in vivo*, at least in the model of cerebral ischemia, whereas the intact CXXC motif is critical in both modes of action of MANF.

## Discussion

In this study, we show that the sequence motif ^148^CXXC^151^ is critically important for the intracellular survival-promoting activity of MANF in cultured peripheral neurons, and it is also required for the neuroprotective activity of extracellularly applied MANF *in vivo*. On the other hand, we show that the ^176^RTDL^179^ motif is not important for the activity of extracellularly applied MANF *in vivo*, but in cultured peripheral neurons the motif is required for antiapoptotic activity insofar as the ER localization of intracellular MANF depends on it.

We found that mutation of the CXXC motif completely abolishes the survival-promoting activity of MANF, both *in vitro*, in the cultured apoptotic SCG and DRG neurons and *in vivo*, in the rat model of stroke. The cysteines of this motif and their position in the loop connecting the helices *α*7 and *α*8 are conserved in the MANF sequences of different species and also in the paralogous protein CDNF. Notably, a corresponding mutation in the MANF of *Drosophila melanogaster* also completely abrogates its ability to rescue MANF mutant larvae from death.^21^ Clearly, the CXXC motif is critically important for the proteins of MANF/CDNF family. However, its precise role remains unknown. The majority of the MANF-C151S protein was located to the ER just as wt MANF, and this localization did not change in the apoptotic conditions, although we cannot exclude fine differences in the localization of MANF and MANF-C151S in the ER. Also, we did not detect any oxidoreductase activity of MANF when using the commonly used biochemical assay of insulin reduction, although we cannot exclude the oxidoreductase activity of MANF on substrates other than insulin, or the requirement of some cofactors that are absent in our biochemical assay. Signaling via free thiol groups of the cysteines in the CKGC motif^30, 31^ cannot be excluded either, but as MANF is mainly found in the ER, the oxidizing environment there should favor the formation of disulfide bonds. Our preliminary nuclear magnetic resonance data indicate that the structural integrity of MANF is not disrupted by the mutation (these data will be published elsewhere). Therefore, we currently favor the explanation that this motif is important for achieving a conformation or structural stability of MANF that in turn could be required for its interactions underlying the prosurvival effect.

It was suggested, based on studies using non-neuronal cells, that ER is the protective *locus operandi* for MANF, but it was not shown that keeping MANF out of the ER renders it nonprotective.^13, 14, 15^ Our data provide evidence that intracellularly MANF protects the SCG neurons in the ER. Indeed, we did not observe major redistribution of MANF from ER to other subcellular locations in the apoptotic conditions where neurons are rescued from the death by MANF. We cannot exclude that a small amount of MANF outside the ER can also be neuroprotective; however, when retrieval of MANF to the ER was abrogated by deletion of the RTDL motif in SCG neurons, the protein accumulated to the Golgi and lost its survival-promoting activity. In the DRG neurons, MANF was unexpectedly retained in the ER even without the RTDL sequence. Why deletion of RTDL did not affect the ER localization of MANF in DRG neurons is currently difficult to explain and remains to be studied. However, the fact that the ER-located MANF-ΔRTDL mutant was neuroprotective in the DRG neurons strongly strengthens our conclusion that the RTDL motif contributes to the survival-promoting activity of MANF by retaining it in the ER. Therefore, although other explanations of our data are possible, the most straightforward one is that MANF can protect the neurons in the ER but not in the Golgi. Interestingly, the MANF of *Drosophila melanogaster*, lacking the corresponding C-terminal RSEL sequence, also partially lost its ability to rescue the lethality of the MANF mutant flies when expressed in a restricted manner.^21^ Although rescue of the whole organism and *in vitro* apoptotic neurons are different processes, the ER localization of MANF seems to be essential in both cases.

The C-terminal RTDL sequence is similar to the classical ER retention signal KDEL and, as shown recently, is indeed able to retain MANF in the ER of non-neuronal cells.^15, 16, 25^ Our data show that RTDL motif behaves as an ER retention signal also in the SCG neurons, as in its absence MANF accumulates to the Golgi. As reported for other cells,^15, 16, 17, 25^ the lack of RTDL motif also increases the secretion of MANF in our SCG model, albeit not massively. However, as our immunolocalization analysis showed, the role of the RTDL motif is clearly different in DRG neurons, where MANF is localized to the ER even in its absence. Whether MANF is retrieved in DRG through other motifs or does not exit from the ER is currently unclear. Our data, from experiments with these two neuronal types, indicate that the mechanisms of MANF trafficking can be cell-type specific, cautioning not to draw generalized conclusions when only one cell type is studied.

MANF and CDNF have both the N-terminal signal peptide^6^ and C-terminal ER retention motif, suggesting that they can be retained in the ER, and, in some conditions, also secreted like classical NTFs. Of note, the other NTFs, as well as all classical growth factors, mitogens, morphogens, peptide hormones, and so on, which act as secreted extracellular factors, do not possess such ER retention motif. MANF and CDNF thus differ considerably from the classical extracellular signaling proteins. It was recently reported that in the cultured cells, KDEL receptors were important for keeping MANF in the intracellular compartment and also increased the levels of MANF in the plasma membrane fractions after ER calcium depletion, suggesting that they may function as MANF receptors both at the Golgi and plasma membrane.^16, 17^ Indeed, Henderson *et al.*^16^ indicated that both KDEL receptors and MANF are trafficking between the surface and the ER. Thus, KDEL receptors may be involved in the mediation of the neuroprotective and neurorestorative effects of MANF *in vivo*.^6, 7, 8, 9, 10, 29, 32^ We wished to test this idea using our MANF-ΔRTDL protein, which is unable to bind the KDEL receptors. Our data show that the C-terminal RTDL sequence is not required for MANF to protect cortical neurons in a model of stroke. How MANF without RTDL motif still protects the neurons is currently unknown. It is also possible that MANF protein, applied extracellularly *in vivo*, protects the neurons via non-receptor mechanisms. We would also interpret the *in vivo* results with caution in terms of generalizing the phenomenon to all neuronal stress situations. Acute ischemic injury causes rapid neuronal damage, whereas in models with chronic stress, such as models of chronic neurodegeneration disorders, the effects of RTDL deletion could be different.

As C-MANF is structurally homologous to the SAP domain of Ku70, reported to bind and inactivate proapoptotic protein Bax,^33, 34, 35, 36^ we raised the hypothesis that intracellular MANF could also prevent cell death via blockage of Bax.^19^ However, using various experimental techniques, including mutation of the putative Bax-binding residues, co-immunoprecipitation in various conditions, surface plasmon resonance, split luciferase complementation assay and different pull-down approaches, we did not find any evidence for such interaction (data not shown). Thus, although the Bax interaction hypothesis cannot be rejected completely, we have not found any evidence that would support it.

In summary, we conclude that intracellularly MANF protects peripheral neurons as a resident ER protein, and in SCG neurons both the CKGC motif and the C-terminal RTDL motif are required for this activity. Notably, the RTDL motif is not required for protection in DRG neurons where it apparently does not contribute to the ER localization of MANF. When applied into the extracellular space of the cerebral cortex, MANF requires the CXXC but not RTDL motif to protect the cortical neurons from ischemic injury. Whether the extracellularly applied MANF acts via cell surface receptors, via ER, or still in a different manner remains to be studied.

## Materials and Methods

### Cloning and expression plasmids

Expression plasmid for human MANF has been described.^19^ MANF-C151S and MANF-ΔRTDL were generated by site-directed inverse PCR mutagenesis using MANF in pCR3.1 vector (Invitrogen, Eugene, OR, USA) as a template. All constructs in pCR3.1 were verified by sequencing with T7 primer, and constructs in pCMV2 were verified by sequencing with T3 primer. All inserts can be excised with *Bam*HI and *Xho*I. The following primers were used: MANF-C151S forward – 5′-GCAAAGGCTCTGCAGAAAAGTC-3′ and MANF-C151S reverse – 5′-ATGTCTCCCCCCAGTCATCCAG-3′ MANF-ΔRTDL forward – 5′-TAGAAGCCGAATTCTGCAGATATC-3′. All constructs produced the proteins of correct size, as verified by western blot on the transiently transfected cells (not shown).

### Neuronal culture and microinjections

Embryonic DRG neurons, from E16 embryos, and neonatal sympathetic SCG neurons were prepared from NMRI mice. The neurons were cultured on polyornithine-laminin- (Sigma-Aldrich, St. Louis, MO, USA) coated dishes or glass coverslips in the Neurobasal medium containing B27 supplement (Invitrogen) and 30 ng/ml mouse 2.5 S NGF (Promega, Madison, WI, USA). SCG neuron cultures were maintained with NGF for 5–6 days, and then microinjected with plasmid DNA and treated with etoposide (Sigma-Aldrich) at 20 *μ*M. DRG neurons were cultured with NGF for 6 days, microinjected with plasmid DNA and treated with 500 nM thapsigargin (Tocris, Bristol, UK; no. 1138) or 20 *μ*M etoposide. Microinjection was performed as published.^19^ On average, for survival assays 50–80 neurons were successfully injected per experimental group in each repeat.

### Immunocytochemistry

The neurons were cultured on glass coverslips and microinjected after 7–12 days *in vitro* with pCR3.1 plasmid encoding for wt human MANF or its mutants. DNA concentration of 5 ng/*μ*l was used. The cells were fixed with 4% PFA at ~24 h (or 48 h for etoposide-treated neurons) after microinjection and stained with the following antibodies (1 : 400 dilution used for all): rabbit anti-MANF (used in Lindholm *et al.*^37^), mouse anti-PDI (Enzo Life Sciences, Farmingdale, NY, USA; no. ADI-SPA-891-F), mouse anti-GM130 (BD Biosciences, San Jose, CA, USA; no. 610823), goat anti-GRP78 (Santa Cruz Biotechnology Inc., TX, USA; no. sc-1051), Alexa Fluor 488 goat anti-rabbit IgG (H+L) (Invitrogen; A-11008) and Alexa Fluor 568 goat anti-mouse IgG (H+L) (Invitrogen; A-11004). The nuclei were stained with DAPI (Sigma-Aldrich; no. D9542). The fluorescent image stacks were acquired using the confocal microscope TCS SP5 equipped with LAS AF 1.82 (Leica Microsystems Inc., Buffalo Grove, IL, USA). The objective was Leica HCX PL APO x63/1.3 GLYC CORR CS (21 °C). The lasers used were DPSS 561 nm/20 mW, OPSL 488 nm/270 mW and diode 405 nm/50 mW, with the beam splitter QD 405/488/561/635. The images were deconvoluted by AutoQuant Auto-Deblur 3D Blind Deconvolution software (Media Cybernetics, Rockville, MD, USA). Deconvoluted images were all processed identically with ImageJ program. For studying the localization of MANF in stressed/dying cells, sympathetic neurons were treated with 20 *μ*M etoposide for 48 h before being fixed.

### Biochemical assays

The ability of recombinant human MANF protein (purified from *E. coli*) to reduce insulin was tested using the Proteostat PDI Assay Kit (Enzo Life Sciences; no. ENZ-51024-KP002) according to the manufacturer's instructions. The insulin-reducing activity of MANF (final concentration 200 nM) and its ability to modulate the activity of PDI were tested either in the presence of DTT or a mixture of GSH (800 *μ*M) and GSSG (200 *μ*M) (Sigma-Aldrich).

### Expression and purification of MANF, MANF-C151S and MANF-ΔRTDL proteins

The MANF, MANF-C151S and MANF-ΔRTDL open reading frames, excluding the signal peptide, were cloned into a T7lac-based vector containing an N-terminal His-tag fusion.^38, 39^ Expression was carried out in the Origami B cells (Novagen, Darmstadt, Germany) in the presence of isopropyl-β-d-thiogalactopyranoside for 3 h at 37 °C. The cells were lysed in the L-buffer (20 mM Tris-HCl, pH 8.0, 0.5% Triton X-100, 0.4 mM phenylmethylsulfonyl fluoride) by sonication, and then NaCl and imidazole were added to final concentrations of 0.5 and 0.02 M, respectively. The lysate was centrifuged (15 000 × * g*, 15 min at 4 °C), and the obtained supernatant was passed through a 0.45 *μ*m filter. The proteins were purified by the HisTrap Kit according to the manufacturer's instruction (GE Healthcare, Buckinghamshire, UK). The buffer of the eluted proteins was exchanged (20 mM phosphate buffer, pH 8.0, 150 mM NaCl) by a PD-10 column. The His-tag was cleaved by AcTEV (Invitrogen) in the absence of DTT. The cleaved products were passed through the HiTrap Chelating column to get rid of the His-tag and AcTEV, whereas the cleaved proteins were obtained in the flow through. The final purification of the proteins was carried out by the HiTrap Q column (GE Healthcare). Buffer exchange and concentration was carried out by using the Amicon Ultra-4 filter device (Millipore, Bedford, MA, USA), and aliquots were stored at –80 °C.

### Intracerebral injections and MCA ligation

Sprague–Dawley rats were anesthetized with chloral hydrate (0.4 g/kg, intraperitoneally). In the first experiment, MANF (*n*=19) or MANF-ΔRTDL (*n*=18) proteins (3 or 6 *μ*g in total) or PBS (*n*=15) were given intracerebrally into two cortical sites ~20 min before MCA occlusion.^40^ In the second experiment, either MANF-C151S (*n*=11) or PBS (*n*=9) were administered in the same manner. Three microliters of MANF solution (0.5–1 *μ*g/*μ*l) or PBS was injected at a rate of 0.5 *μ*l/min at each site. The needle was retained in place for 5 min after injection. Ligation of the right MCA and bilateral common carotids (CCAs) was performed by using methods described previously.^41^ Briefly, the bilateral CCAs were identified and isolated through a ventral midline cervical incision. Rats were placed in a stereotaxic apparatus, and a craniotomy was made in the right hemisphere. After the past injection, the right MCA was ligated with a 10-0 suture and bilateral CCAs were ligated with nontraumatic arterial clamps for 60 min. After 60 min of ischemia, the suture around the MCA and arterial clips on CCAs were removed. After recovery from anesthesia, the rats were returned to their home cage. The infarction area was measured by TTC staining two days after MCA occlusion, as described previously.^9^

## Abbreviations

MANF: mesencephalic astrocyte-derived neurotrophic factor
C-MANF: carboxy-terminal domain of MANF
wt MANF: wild-type MANF
CDNF: cerebral dopamine neurotrophic factor
DRG: dorsal root ganglion
NTF: neurotrophic factor
SCG: superior cervical ganglion
ER: endoplasmic reticulum
PDI: protein disulfide isomerase
DTT: dithiothreitol
MCA: middle cerebral artery
TTC: triphenyltetrazolium chloride
PBS: phosphate-buffered saline
UPR: unfolded protein response
