# Supplementary material for: Role of two sequence motifs of mesencephalic astrocyte-derived neurotrophic factor in its survival-promoting activity
Source: Cell Death Dis. 2015 Dec 31;6(12):e2032–. doi: 10.1038/cddis.2015.371 (PMC4720903; doi:10.1038/cddis.2015.371)
Supplement: Supplementary Figure S1 [file cddis2015371x1.pdf]

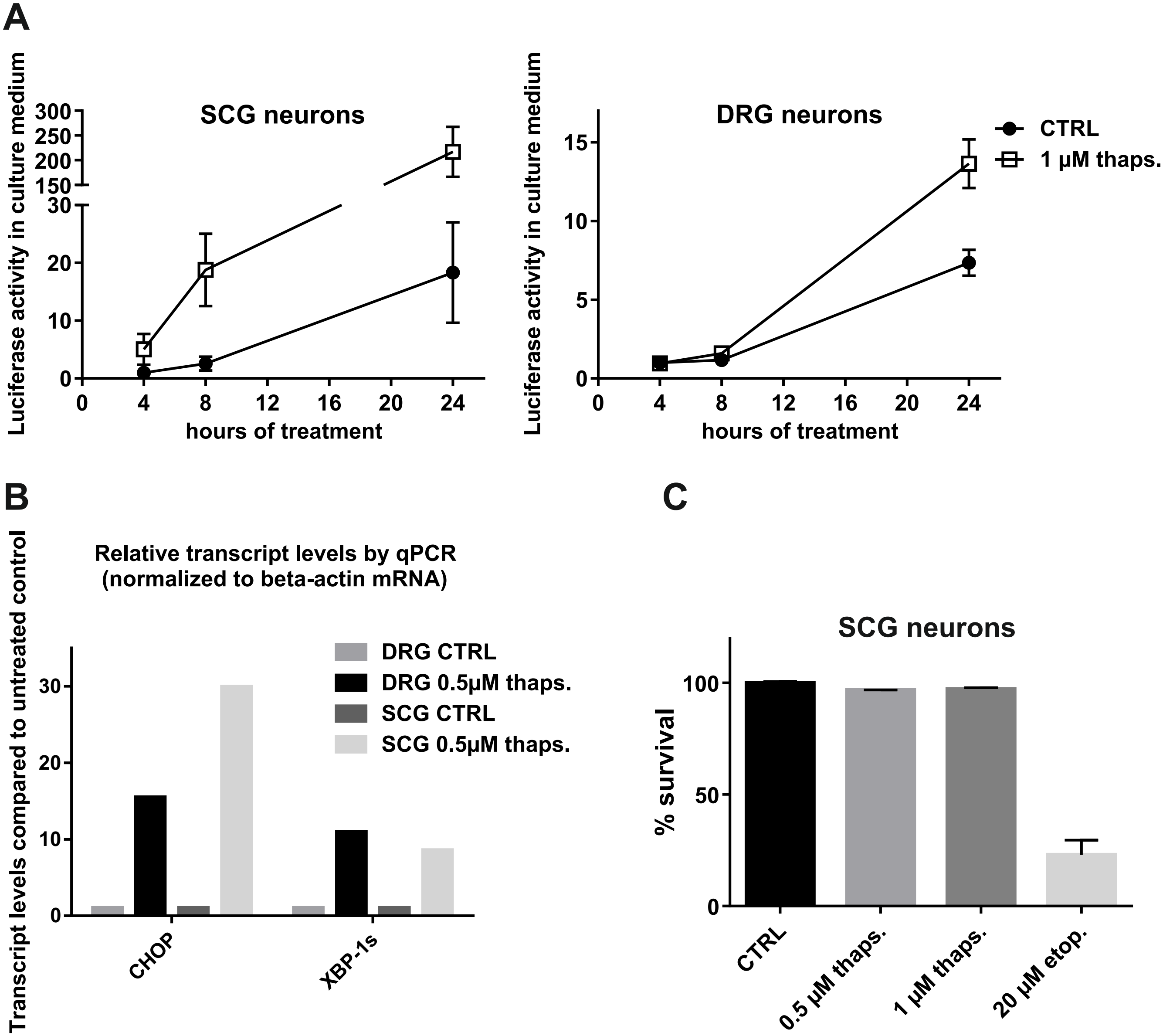

Supplementary Figure 1. (**A**) Thapsigargin treatment causes ER Ca^{2+} depletion in both DRG and SCG neurons. E16 DRG neurons and neonatal SCG neurons were cultured in the presence of NGF and on day 5 or day 3, respectively, transduced with adeno-associated virus vector (dsAAV serotype 1) carrying the SERCaMP reporter which has luciferase activity and is secreted into culture medium upon ER Ca^{2+} depletion (Henderson MJ et al., Mol Biol Cell. 2014 Sep 15; 25(18):2828-39). 3 days later the cells were treated with 1µM thapsigargin and the culture medium was sampled 4, 8 and 24 hours after the start of treatment. The graphs present average luciferase activity measured from 2 to 3 replica wells in a single experiment (means ± SD is shown). The values are expressed as fold difference from reporter activity in untreated control wells at 4h. Before the viral transduction the cultures were pulsed with 5'-fluorodeoxyuridine and uridine to eliminate non-neuronal cells. 5µM pan-caspase inhibitor Q-VD-OPh was present during the treatment to prevent possible reporter release resulting from cell death and rupture. (**B**) Thapsigargin treatment causes unfolded protein response in both DRG and SCG neurons. E16 DRG neurons and neonatal SCG neurons were cultured in the presence of NGF and were treated with 0.5µM thapsigargin on day 7 and day 5, respectively. RNA was extracted 24h later and the mRNA levels of CHOP and XBP-1s isoform were determined by quantitative real-time PCR performed with the LightCycler 480 real-time PCR system (Roche Diagnostics, Basel, Switzerland) using LightCycler 480 SYBR Green I Master. The results were analyzed as described previously (Varendi K et al., Cell Mol Life Sci. 2014 Nov;71(22):4443-56). The primer sequences have been published previously (Zhang W et al., Cell Metab. 2006 Dec;4(6):491-7). The graph presents the transcript/actin mRNA ratio expressed as fold difference from the ratio in untreated neurons. The result of a single experiment is shown. The cultures were pulsed with 5'-fluorodeoxyuridine and uridine to eliminate non-neuronal cells. (**C**) Thapsigargin does not kill SCG neurons. Neonatal SCG neurons were cultured in the presence of NGF and on day 6 or day 7 they were treated with thapsigargin or etoposide. The number of surviving neurons was counted three days later and is expressed as % of their initial number, counted right after the start of treatment. Shown are the means ± s.e.m of three independent experiments (n=3).
