# Supplementary material for: Role of two sequence motifs of mesencephalic astrocyte-derived neurotrophic factor in its survival-promoting activity
Source: Cell Death Dis. 2015 Dec 31;6(12):e2032–. doi: 10.1038/cddis.2015.371 (PMC4720903; doi:10.1038/cddis.2015.371)
Supplement: Supplementary Figure S2 [file cddis2015371x2.pdf]

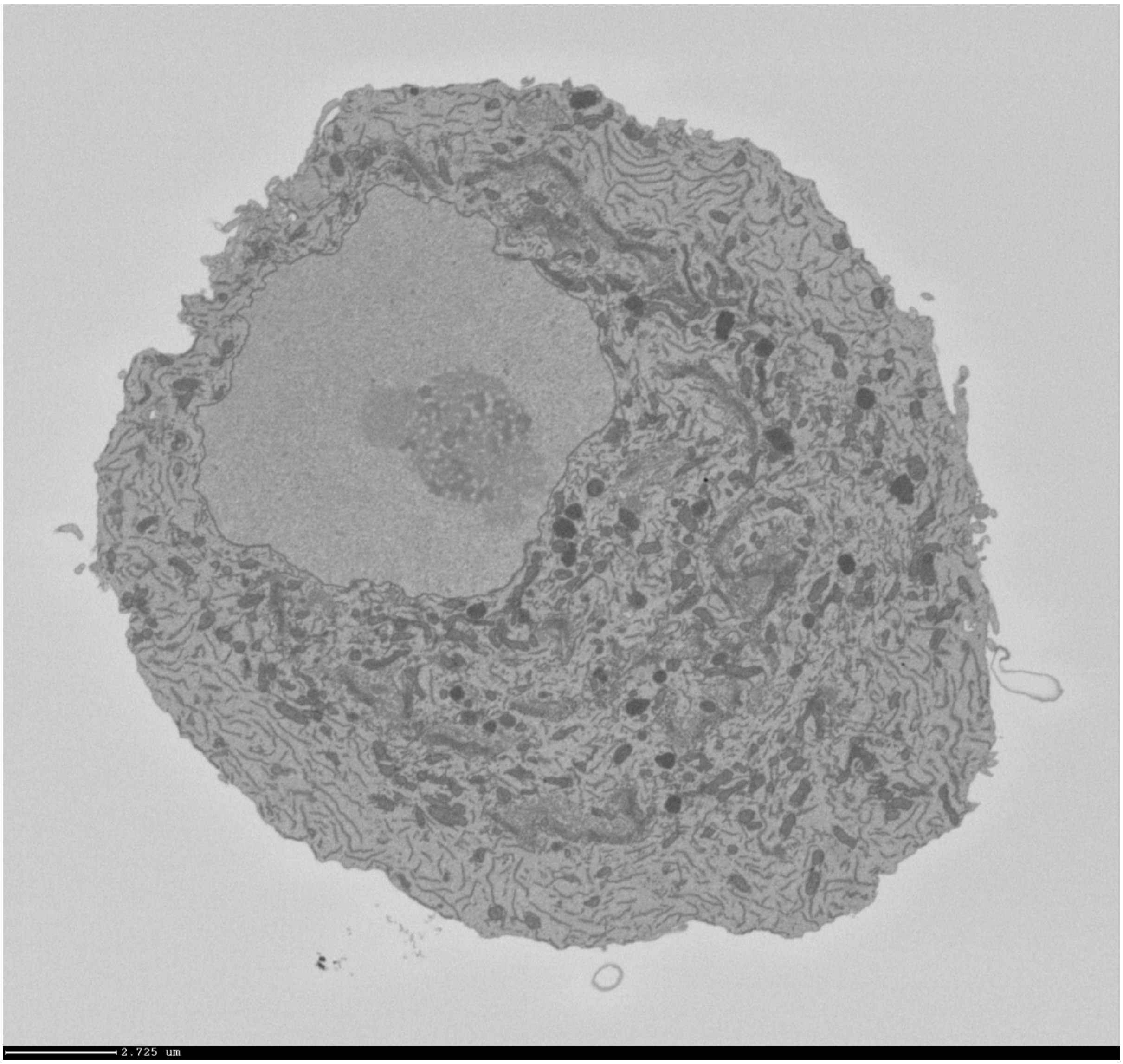

Supplementary Figure 2. Block face image of the sympathetic neuron from newborn mouse superior cervical ganglion (SCG), grown on glass coverslip for six days with nerve growth factor (NGF), was acquired with a FEG-SEM Quanta 250 (FEI) using a backscattered electron detector (Gatan). Specimen preparation and imaging conditions as described in Puhka et al., Mol. Biol. Cell 23:2424-2432, 2012. Note the densely packed endoplasmic reticulum on the periphery of the cell. The scale bar is shown on the left bottom corner.
