# Supplementary material for: Role of two sequence motifs of mesencephalic astrocyte-derived neurotrophic factor in its survival-promoting activity
Source: Cell Death Dis. 2015 Dec 31;6(12):e2032–. doi: 10.1038/cddis.2015.371 (PMC4720903; doi:10.1038/cddis.2015.371)
Supplement: Supplementary Figure S3 [file cddis2015371x3.pdf]

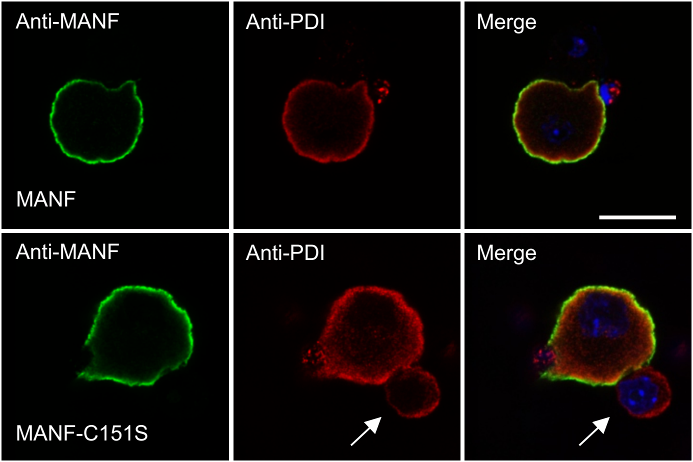

Supplementary Figure 3.
Immunolocalization of overexpressed MANF or MANF-C151S in healthy DRG neurons. NGF-maintained DRG neurons were microinjected with the expression plasmids for MANF or MANF-C151S and grown in the presence of NGF for 24 h. The cultures were co-stained with antibodies to MANF (green) and the ER marker PDI (red). Nuclei were labeled with DAPI (blue). Shown are the confocal microscopic images of typical expression patterns. The MANF and MANF-C151S always co-localized with the ER marker. Note the absence of MANF immunoreactivity in the uninjected neuron in the middle row of the MANF panel (arrow), showing the absence of non-specific back-ground and undetectable levels of endogenous MANF. Scale bar, 12 μm.
