# Supplementary material for: Role of two sequence motifs of mesencephalic astrocyte-derived neurotrophic factor in its survival-promoting activity
Source: Cell Death Dis. 2015 Dec 31;6(12):e2032–. doi: 10.1038/cddis.2015.371 (PMC4720903; doi:10.1038/cddis.2015.371)
Supplement: Supplementary Figure S4 [file cddis2015371x4.pdf]

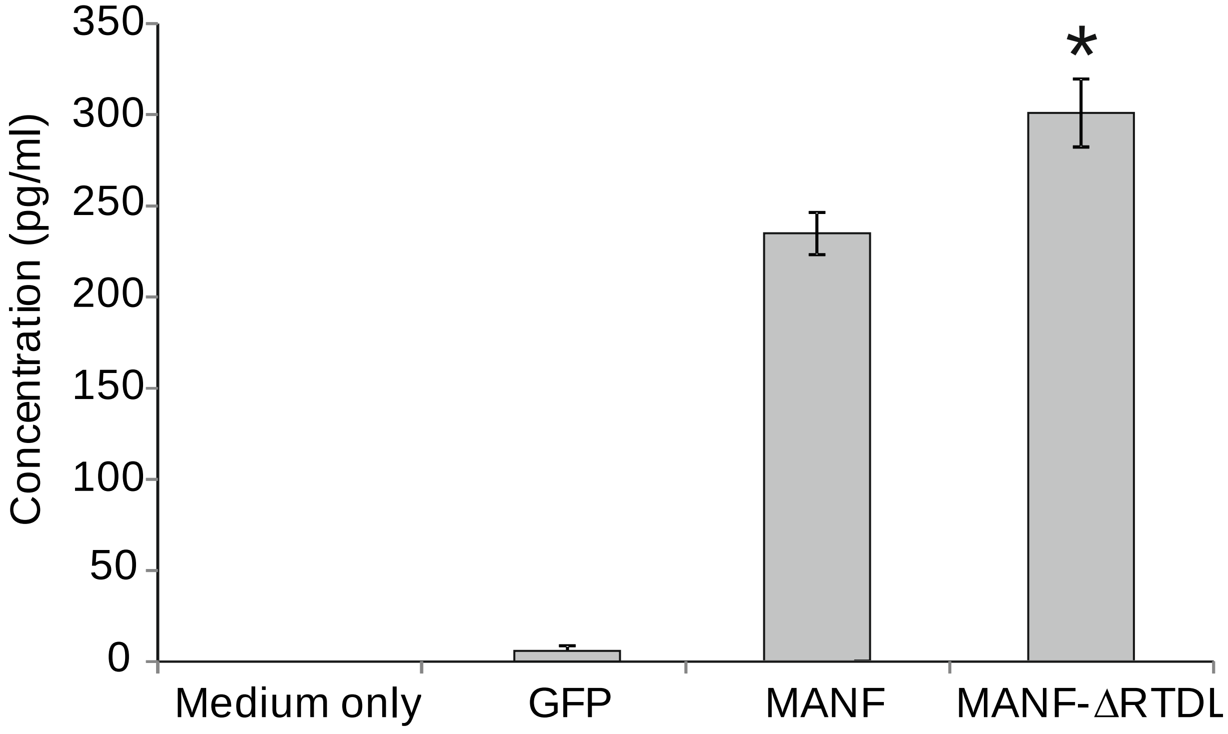

Supplementary Figure 4. Determination of the levels of MANF and MANF-ΔRTDL in the culture media of microinjected SCG neurons. 7-days old NGF-maintained mouse SCG neurons were microinjected with the plasmids for MANF or MANF-ΔRTDL together with the plasmid for GFP, or only for GFP as a control. About 450-500 neurons were injected per experimental point. After 24 h, the culture media were collected and concentrated with Amicon Ultra-4 centrifugal filter units (Merck Millipore) to 200 μl. The levels of MANF and MANF-ΔRTDL were measured by sandwich Enzyme-Linked Immunosorbent Assay (ELISA). A detailed protocol for the MANF-ELISA will be published elsewhere (Galli E, Lindholm P et al. unpubl.). Briefly, 96-well flat-bottom microtiter plates (Thermo Scientific, #430341) were coated with anti-human-MANF antibody diluted in 0.05 M carbonate buffer, pH 9.6 for 24 h. After washing, the wells were blocked with PBS containing 0.05% of Tween 20 and 1% of casein, then incubated with the samples of the culture media or the known amounts of recombinant proteins at +4°C overnight, followed by incubation with peroxidase-conjugated anti-MANF antibody diluted in the blocking solution for 5 h at room temperature. The reaction was developed by DuoSet ELISA Development System (R&D) and visualized at 450 nm. The assay was repeated twice. Protein concentrations in the media were calculated from the standard curves of MANF, determined for each assay. *Post hoc* analysis followed by one-way ANOVA revealed significant difference between MANF and MANF-ΔRTDL, indicated by *, whereas no MANF was detected from the control medium without cells or in the medium from the GFP-expressing control neurons.

The accuracy of the assay was demonstrated by its ability to recover the known amounts of recombinant MANF and MANF-ΔRTDL proteins, as determined from the standard curve (Table 1). This test also shows that the assay recognizes both proteins with similar efficiency.

Table 1.

**Input (pg/ml)**	**Recovery (pg/ml)**
MANF (1000)	1295
MANF (500)	495
MANF-ΔRTDL (1000)	1417
MANF-ΔRTDL (500)	548
PBS	25
